# Validation of the Functional Assessment of Cancer Therapy with Cervical Cancer Subscale (FACT-CX) for Quality of Life in Thai Patients Prior to Chemoradiotherapy

**DOI:** 10.31557/APJCP.2020.21.7.1891

**Published:** 2020-07

**Authors:** Thanarpan Peerawong, Yuthasak Suphasynth, Chanon Kongkamol, Paytai Rordlamool, Jidapa Bridhikitti, Duangjai Sangtawan, Rungarun Jiratrachu, Thiti Atjimakul, Saibua Chicharoen

**Affiliations:** 1 *Department of Radiology, Faculty of Medicine, Prince of Songkla University, Songkhla, Thailand. *; 2 *Department of Obstetrics and Gynecology, Faculty of Medicine, Prince of Songkla University, Songkhla, Thailand. *; 3 *Research Unit of Holistic Health and Safety Management in the Community, Faculty of Medicine, Prince of Songkla University Songkhla, Thailand. *

**Keywords:** Validation, FACT, CX, quality of life, cervical cancer

## Abstract

**Objective:**

Cervical cancer is the second most common cancer in Thailand. For cervical cancer, there is no cancer specific quality of life questionnaire. This study aims to develop and validate Thai FACT-CX.

**Methods:**

The cross-sectional study included all women aged ≥18 years with stage IB2-IIIB who planned to undergo chemoradiotherapy. Those who did not understand Thai language, had other cancers (except for skin cancer), were diagnosed with impaired cognition and/or overt psychosis, and major depression were excluded. The FACT-CX comprises 42 items with 5 domains and a score range of 0-168. The WHOQOL-BREF comprises 26 items with 4 domains and a score range of 26-130. The participants were interviewed about demographic and clinical data. Both questionnaires were self-completed. Factor analysis was used to compare our data with the previous structure. The reliability used Cronbach’s alpha. Spearman’s correlation determined relationship between the domains of the modified FACT-CX and WHOQOL-BREF. Both questionnaires were compared with socioeconomic and clinical variables using the Ranksum test and Kruskal-Wallis test. P-value > 0.05 considered significant.

**Results:**

The 245 participants included. Expletory factor analysis revealed an accumulative variance of 0.42 with 4 factors. The internal consistency was 0.84, 0.81, 0.78, 0.77 and 0.90 for perception of self, suffering symptoms, family support, life resilience and total questions. There was correlation between the domains of the modified FACT-CX and WHOQOL-BREF. Both the modified FACT-CX and WHOQOL-BREF could identify differences between the groups of patients.

**Conclusion:**

Finally, the Thai modified FACT-CX was found to be reliable and valid for measuring quality of life among untreated cervical cancer patients.

## Introduction

Cervical cancer is the fourth most common cancer and the fourth leading cause of death in women worldwide; however, it is the second most common cancer in Southeast Asia, including Thailand (Ferlay et al., 2013). In the early stages it is usually asymptomatic, and surgery is the treatment of choice. But when it becomes more invasive, patients will develop massive vaginal hemorrhage, pelvic pain, lower-extremity swelling, and micturition problems. In this situation, chemoradiotherapy is indicated for both curative and palliative reasons (Niederhuber et al., 2014). While waiting for radiotherapy, fear and misperception of radiotherapy cause anxiety (Gillan et al., 2014). Both physical symptoms and emotional distress can affect patient quality of life. 

Quality of life is one of the most important clinical results. A summary of the most commonly used quality of life questionnaires and their details are provided in Table 1 (Tax et al., 2017). The WHOQOL-BREF is a shorter version of the World Health Organization’s quality of life questionnaire, used and validated for the measurement of general health related quality of life (Development of the World Health Organization WHOQOL-BREF quality of life assessment, the WHOQOL Group, 1998). This questionnaire covers a wide range of conditions in order to compare patients with diseases to the general population. However, due to the generic nature of this questionnaire, it does not focus on the issues of particular concern to patients with specific diseases. Therefore, a disease specific questionnaire may be more sensitive and thus detect any differences (Fayers and Machin 2007). 

Nowadays, there are 2 common specific measurements for quality of life in cervical cancer: Functional Assessment of Cancer Therapy with Cervical Cancer Subscale (FACT-CX) and the Cervical Cancer Module (QLQ-CX24) from the European Organization for Research and Treatment of Cancer Quality of Life. FACT-CX is a cervical cancer specific quality of life questionnaire that has been be validated in English, Chinese and Portuguese (Ding et al. 2012; Fregnani et al. 2013). FACT-CX uses 42 items. In contrast, QLQ-CX24 uses 24 items and is more popular, but in practice QLQ-CX-24 was designed to supplement the European Organization for Research and Treatment of Cancer Quality of Life Questionnaire (EORTC QLQ-C30) (Greimel et al. 2006). The total number of questions is 54. Hence, the FACT-CX is shorter and may be more convenient due to its brevity.

To the best of our knowledge, no cervical cancer specific questionnaire has been validated in the Thai language. This study aimed to develop and validate the Thai version of the FACT-CX for measuring quality of life compared with the WHO-BREFF in untreated cervical cancer patients.

## Materials and Methods

*Study design and setting*

The cross-sectional study was performed in the Radiation Oncology Clinic at the largest university hospital in Southern Thailand. There was a mix of Buddhist and Muslim patients. The hospital setting is tertiary care with approximately 2500 new radiotherapy consultations per year from across Southern Thailand. Our treatment policy: radiotherapy is indicated if the disease is locally advanced with post-operative intermediate or high risk. The enrollment period was between February 2014 and March 2016.

*Study samples*

Women with newly diagnosed stage IB2-IIIB cervical carcinoma who were aged more than 18 years and planned to undergo concurrent chemoradiotherapy were included. Those who did not understand the Thai language, had other cancers (except for skin cancer), and were diagnosed with impaired cognition and/or overt psychosis, major depression or delirium were excluded.

*Instruments*

FACT-CX is the Functional Assessment of Cancer Therapy-General (FACT-G) with cervical cancer subscale. The researchers collaborated with the Functional Assessment of Chronic Illness Therapy (FACIT) organization on the translation of the questionnaire. Linguistic validation was also performed with the FACIT organization using its guidelines and process of back translation. 

The final version of the Thai FACT-CX was pilot tested with 10 cervical carcinoma patients using the interview script provided by the organization. We found problems with 2 questions. Firstly, “I am bothered by discharge or bleeding from my vagina;” the word “discharge” is difficult to understand in the Thai language. Then the word “leucorrhea” was added to make the sentence clearer. Secondly, “My vagina feels too narrow or short;” this sentence was doubtful. The issue was, “How do they know their vagina is too short or narrow?” We think this sentence could be understood by sexually active ladies only, or when they or their doctor inserts fingers into their vagina. Some of the ladies were not sexually activity after treatment. They feared sexually activity because of pain or for other reasons. Thus, they could not understand this sentence. The gynecologic nurse and research assistant suggested adding the word “constricted” in order to magnify the understanding of this question.

After adapting some questions with the permission of the FACIT organization, the second pilot study was conducted in 10 different patients, and we found that all the patients understood the translation. 

FACT-CX comprises 42 items with a 5-point (0-4: Not at all to very much) Likert scale and is categorized into 5 domains: physical well-being (PWB), social/family well-being (SWB), emotional well-being (EWB), functional well-being (FWB) and cervical cancer subscales (CCS). The range of scores for these domains was 0-28, 0-28, 0-24, 0-28 and 0-60, respectively. The range of total score for FACT-CX was 0-168. A higher score means a higher quality of life

WHOQOL-BREF was translated into the Thai language in 1998 and validated in radiotherapy patients (Mahatnirunkul et al. 1998; Phungrassami et al. 2004). The radiotherapy patients used 13±4.0 minutes to complete it. The questionnaire comprises 26 items with a 5-point (1-5: not at all to very much) Likert scale is and categorized into 4 domains; physical health (PH), psychological well-being (PSW), social relationships (SR) and satisfaction with the environment (SE). The score of the subscale was calculated by summing the corresponding items in the subscale. The overall score was the sum of all the items and ranged from 26-130. Higher score means higher quality of life. The scores were grouped into bad (26-60), average (61-95) and good (96-130).

The independent variables were demographic and clinical characteristics. Demographic characteristics included age, religious, marital status, education level, child adequacy, economic and working status. The clinical characteristics were clinical stage, Eastern Cooperative Oncology Group (ECOG) Performance Status, had undergone percutaneous nephrostomy, and current symptoms.

*Data collection*

One week after the diagnosis was made by gynecologists and radiation oncologists, all eligible women, based on the inclusion and exclusion criteria, who visited the Radiation Oncology Clinic were provided with information and invited to participate in the study by a trained research assistant. After they signed the consent form, the research assistant interviewed the participants regarding their demographic data. The clinical part was assessed by researchers. The questions on the WHOQOL-BREF and FACT-CX were self-completed by the patients. If the patients had reading difficulties, research assistants would read each item aloud before the patient picked her choice. Total time spent on these procedures was about 30 minutes. 

*Statistical analyses*

Demographic and clinical characteristics were analyzed descriptively. The domains of FACT-CX (PWB, SWB, FWB, EWB and CCS) were checked for data fit using confirmatory factor analysis (CFA). If the model was poorly fitted, exploratory factor analysis (EFA) was performed. The number of factors in EFA was chosen by scree plot in order to have the eigenvalues closest to unity. The acceptable level of loading for each variable was 0.30. For items with inter-correlation above 0.8, the lowest loading score was dropped. Oblique rotation technique (oblimin) was used during factor extraction in accordance with previous studies (Anna and Jason, 2005; Ratanatharathorn et al., 2001). 

The domains identified from EFA were checked for internal reliability using Cronbach’s alpha. For the validation process, Spearman’s correlation was calculated to determine the relationships between the domains of the current version of FACT-CX and of WHOQOL-BREF. Both scales finally had their relationships with the demographic and clinical characteristics compared using the Ranksum test and Kruskal-Wallis test. P-value of less than 0.05 was considered significant.

The sample size was calculated to test the validity of the questionnaire using exploratory factor analysis. An adequate sample in the current practice is a 1:2 to 1:>100 item respondent ratio (Anna and Jason, 2005). We chose a 1:5 item respondent ratio in the study. The total number of items in FACT-CX is 42. Hence, the estimated sample size was at least 210 cases. An additional 15% were added. Thus, a total of 245 participants are included in the study.

The study was approved by the Human Research and Ethics Committee of the Prince of Songkla University, EC number: 56-298-07-1-3.

**Table1:** The Quality of Life Questionnaire

	Questionnaires					
Attribution	SF-36	WHO-BREF	EORTC QLQ-c30	FACT-G	QLQ-CX24	FACT-CX
Designed for	General	General	Cancer	Cancer	Cervical cancer	Cervical cancer
Number of item	36	26	30	27	24	42
Number of study	21	9	29	26	35	22
Validated in Thailand	Yes	Yes	Yes	Yes	No	No

**Figure 1.**
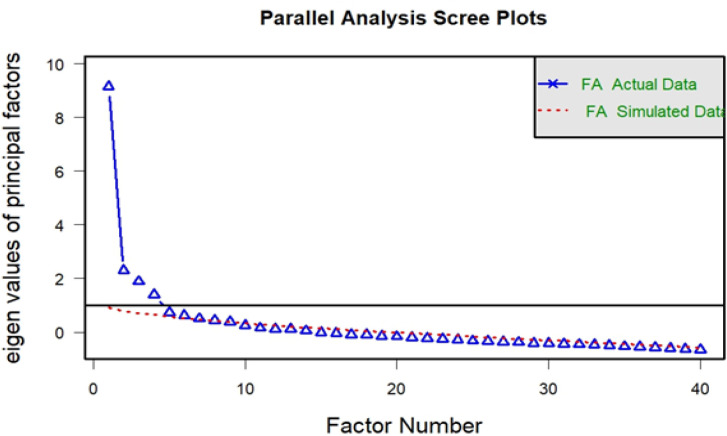
Relationship between Number of Factor and Eigenvalue. FA; Factor analysis

**Figure 2:**
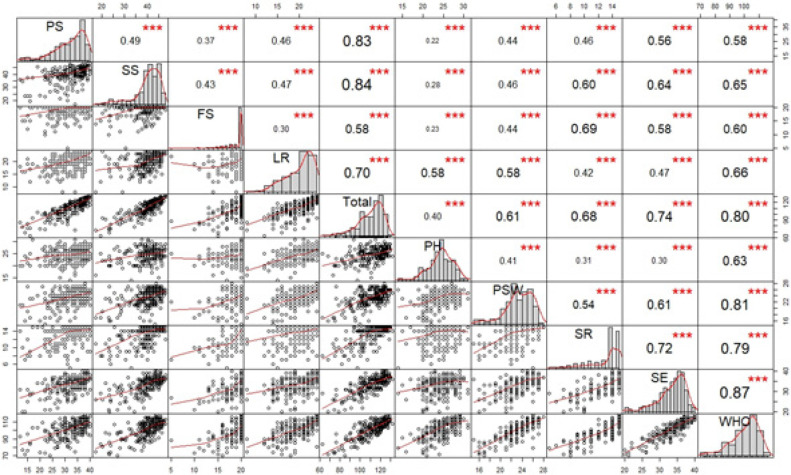
Correlation Matrix between Domain in Modified FACT-CX and WHOQOL-BREF. The statistic calculated by Spearman’s correlation. *** p ≤ 0.001; Abbrivation: PS, Perception to self; SS, Suffering symptom (SS); FS, Phychological well-being; SR, Social relationship; SE, Satisfaction with the environment; WHO, overall score of WHOQOL-BREF

**Table 2:** Sociodemographic and Clinical Patient Characteristics (n=245)

Variables		n (%)
Age (mean and SD)		50.34 ±12
Religious		
	Buddhism	201 (82)
	Islamism	44 (18)
Status		
	Single	9 (3.7)
	Married or couple	181 (73.9)
	Divorce	55 (22.4)
Education level		
	Bachelor and above	21 (8.6)
	Secondary school	53 (21.6)
	Primary school	160 (65.3)
	Unlettered	11 (4.5)
Child adequacy		233 (95)
Economic problem		160 (65.3)
Working		90 (36.7)
Infected with HIV		14 (5.7)
Stage		
	IB2	5 (2)
	II	163 (64..9)
	III	81 (33.1)
ECOG performance status		
	0-1	234 (95.5)
	2-3	11 (4.5)
Nephrostomy		22 (9)

**Table 3:** The Exploratory Factor Analysis of the Modified FACT-CX

	item	PS	SS	FS	LR	h2	u2	com
ge6	I worry that my condition will get worse	0.78				0.59	0.41	1
ge5	I worry about dying	0.72				0.53	0.47	1
ge4	I feel nervous	0.72				0.55	0.45	1.1
cx5	I am afraid the treatment may harm my body	0.6				0.31	0.69	1.1
ge1	I feel sad	0.59				0.46	0.54	1.2
ge3	I am losing hope in the fight against my illness	0.59				0.34	0.66	1.1
gf4	I have accepted my illness	0.56				0.5	0.5	1.6
gf6	I am enjoying the things I usually do for fun	0.51			0.4	0.63	0.37	2
ge2	I am satisfied with how I am coping with my illness	0.49				0.47	0.53	1.6
gf7	I am content with the quality of my life right now	0.41				0.34	0.66	1.7
gp6	I feel ill		0.62			0.45	0.55	1.2
bl1	I have trouble controlling my urine		0.54			0.41	0.59	1.3
cx2	I am bothered by odor coming from my vagina		0.54			0.3	0.7	1.4
cx7	I have discomfort when I urinate		0.53			0.41	0.59	1.9
gs7	I am satisfied with my sex life		0.53			0.32	0.68	1.1
cx1	I am bothered by discharge or bleeding from my vagina		0.53			0.28	0.72	1.1
cx4	My vagina feels too narrow or short		0.53			0.28	0.72	1.1
gp1	I have a lack of energy		0.49		0.34	0.47	0.53	1.8
cx6	I am bothered by constipation		0.46			0.28	0.72	1.2
gp5	I am bothered by side effects of treatment		0.39			0.31	0.69	1.9
bl3	It burns when I urinate		0.39			0.34	0.66	2.8
gp4	I have pain		0.36			0.21	0.79	2.2
gs5	I am satisfied with family communication about my illness			0.84		0.7	0.3	1
gs4	My family has accepted my illness			0.72		0.58	0.42	1.1
gs2	I get emotional support from my family			0.72		0.53	0.47	1
gs6	I feel close to my partner (or the person who is my main support)			0.61		0.41	0.59	1
gs3	I get support from my friends		0.36	0.42		0.46	0.54	2.1
c6	I have a good appetite				0.83	0.66	0.34	1
hn1	I am able to eat the foods that I like				0.76	0.58	0.42	1
gf3	I am able to enjoy life	0.38			0.48	0.54	0.46	2
gf2	My work (include work at home) is fulfilling				0.44	0.34	0.66	1.5
gp2	I have nausea				0.38	0.25	0.75	2.4
c7	I like the appearance of my body				0.36	0.18	0.82	1.2
	Cumulative variance 0.42	0.13	0.11	0.09	0.09			

PS, Perception to self; SS, Suffering symptom; FS, Family support; LR, life resilience

**Table 4.** Scoring Method and Cronbach’s Alpha Coefficients of Modified FACT-CX

Subscales	Items	Score range	Cronbach’s alpha
Perception to self	10	0-40	0.87
Suffering symptom	12	0-48	0.81
Family support	5	0-20	0.78
Life resilience	6	0-24	0.77
Total score	33	0-132	0.9

**Table 5:** The Quality of Life Score of WHOQOL-BREF and Modified FACT-CX Classified by Patient Charectoristics (N=245)

Patient charecteristics		Quality of life score			
		WHOQOL-BREF		Modified FACT-CX	
		Median (IQR)	*P*-value	Median (IQR)	*P*-value
Age (years)	≥60 (17.1%)	105 (101.5,109.8)	0.102	122 (115.3,125.6)	< 0.001
	<60 (62.9%)	104 (96,109)		113.8 (101.9,120.5)	
Working	Yes (36.7%)	105 (96,109)	0.999	115.6 (104.1,120.9)	0.72
	No (63.3%)	104 (97,109)		115.2 (103.9,122.2)	
Economic problem	Yes (65.3%)	103 (95,108)	0.004	113.5 (102.3,120.5)	0.006
	No (34.7%)	107 (99,111)		118 (107,124.2)	
Stage	Ib2 (2.0%)	105 (102,107)	0.568	119.2 (114.2,122.8)	0.384
	II (64.9%)	105 (97,109)		115.5 (105.1,122.2)	
	III (33.1%)	103 (96,109)		114.5 (101.2,120.5)	
ECOG	0-1 (95.5%)	105 (97,109)	< 0.001	115.8 (105,122.2)	< 0.001
	2-3 (4.5%)	91 (88,97)		100.2 (94,103.4)	
Percutaneous nephrostomy	Yes (9.0%)	89.5 (86.2,98.8)	< 0.001	102.1 (92.3,113.4)	< 0.001
	No (91.0%)	105 (98,109)		115.8 (105.1,122.3)	
Symptom					
Fatigue	Yes (33.9%)	101 (94,107)	0.001	108.2 (101,116.5)	< 0.001
	No (66.1%)	105 (99,109)		118.8 (108.4,123.5)	
Vaginal hemorrhage	Severe (2%)	95 (94,98)	0.261	108.2 (101.8,116.5)	0.452
	Moderate (5.7%)	100 (94.8,107.5)		107.1 (101.5,123.1)	
	Mild (38.4%)	104.5 (96,109)		114.8 (104.6,122.2)	
	No (53.9%)	105 (97.8,109)		116.5 (105,121.8)	
Vaginal discharge	Moderate to heavy (18.8%)	100 (90.2,107)	0.039	108.5 (100.3,118.6)	0.02
	Mild (40.4%)	105 (98,109.5)		116.2 (107.1,121.6)	
	No (40.8%)	105 (97,109)		116.1 (104.3,123)	
Pelvic pain	Severe pain (2.4%)	88.5 (84.8,90)	0.006	87.9 (82.8,97.8)	< 0.001
	Moderate pain (10.2%)	103 (92,109)		109.2 (94.5,116.8)	
	Mild pain (38.0%)	105 (98,109)		115.2 (106.5,121.8)	
	No (49.4%)	105 (98,109)		116.5 (105,122.8)	
Urinary incontinence	Yes (6.1%)	89 (82,94)	0.083	106.8 (98.8,116.8)	0.052
	No (93.9%)	105 (97,109)		115.5 (104.8,122.2)	
Quality of life	Good (78.4%)	107 (102,110)	< 0.001	118.5 (110.8,123.1)	< 0.001
	Fair ( 21.6%)	89 (84,92)		98.8 (86,103.8)	

The statistic calculated by Ranksum test and Kruskal-Wallis test

## Results

*Characteristics of the subjects*

Of the 245 participants, the majority were married, middle-aged Buddhist women with only a primary education and economic problems. Distribution of disease stages was IB (2%), II (64.9%) and III (33.1%). Nine percent had undergone nephrostomy and 5.7% were HIV positive (Table 2).

*Factor analysis*

Initially, the CFA revealed that our data did not fit the previous structure. The p-value from the Chi-square test (809 degrees of freedom) was < 0.001. The comparative fit index (CFI) was 0.573 and the Tucker-Lewis index (TLI) was 0.546. The root mean square of approximation (RMSA) was 0.13 and the standardized root mean square residual (SRMR) was 0.098. All these statistics indicate that our data poorly fit the construct proposed by the previous study. 

In EFA, the Kaiser-Meyer-Olkin of sampling adequacy was 0.82 and the Bartlett’s test of sphericity was significant with p-value <0.001. The details of EPA results are shown in Table 3. The cumulative variance was 0.42. Loading of the variables for each factor ranged from 0.36-0.84. The loading of factor 1, factor 2, factor 3 and factor 4 were 4.44, 3.73, 2.97 and 2.86, respectively. The 4 new factors were: perception to self (PS), suffering from symptoms (SS), family support (FS) and life resilience (LR). Total items were reduced from 42 to 33 questions.

The Cronbach’s alpha in each domain and the total items of the modified FACT-CX ranged from 0.77-0.90 (Table 4). Internal consistency of the total items was excellent. There was good internal consistency in the PS and SS domains, but only acceptable internal consistency in the FS and LR domains.

*Validity*

The convergent validity of the modified FACT-CX and WHOQOL-BREF are shown in Figure 2. The PS domain had a moderate correlation with SE with a correlation coefficient of 0.56 for the WHOQOL-BREF. The p-value was >0.001. The SS domain had a moderate correlation with the SR and SE domains; the correlation coefficients were 0.60 and 0.64, respectively. The p-value was >0.001. The FS domain had a moderate correlation with SR and SE. The correlation coefficients were 0.69 and 0.58 with p-value >0.001. The LR had a moderate correlation with the PH and PSW domains. The total score of the modified FACT-CX had a high correlation with the total score of the WHOQOL-BREF. The correlation coefficient was 0.80 (p >0.001).

The findings of quality of life measured by WHOQOL-BREF and the modified FACT-CX were quiet similar. The questionnaire could identify the differences between the groups of patients with economic problems, ECOG performance status, percutaneous nephrostomy, fatigue, level of vaginal discharge, and severity of pelvic pain. The modified FACT-CX could identify the differences in quality of life score between patients aged more than 60 years and less than 60 years. When comparing the modified FACT-CX with the classified WHOQOL-BREF, the modified FACT-CX could categorize quality of life into good and average, with reference to the normal population.

## Discussion

The Thai modified FACT-CX is a cervical cancer specific quality of life questionnaire. It has 33 items, including 4 domains. The questionnaire had excellent internal consistency. In construct validity, the questionnaire had a high correlation and the same differentiation of demographics as the WHOQOL-BREF. With the exception of aged more than 60 years, the Thai modified FACT-CX could detected different of quality of life score. The original version had 42 items, including 5 domains. However, the Thai modified FACT-CX had a loose ability of FACT-G when comparing quality of life with the other cancers. There are 2 developed and validated studies of FACT-CX. Ding et al.,’s (2012) study included 400 Chinese women with cervical cancer and assessed the EFA with 4 factors; the cumulative variance was 0.50. However, the variance included only the variables of the PWB, SWB, FWB and EWB. Another study, by Fregnani et al., (2013) included 100 participants. However, this study used an item per respondent ratio of 1:2.4 and did not report the Kaiser-Meyer-Olkin sampling adequacy or Bartlett’s test of sphericity. The suitability of samples for performing EFA is unknown. With the 5 forced factors, the cumulative variance was 0.46 and loading was not the same as in the previous structure. Therefore, comparing our result to the previous study is difficult. 

However, the reliability in the structures of the modified FACT-CX was at least acceptable. The Chinese study had poor internal consistency in the CCS domain because of bmt7, “I have concerns about my ability to have children.” This question had little significance in cervical cancer patients in Mainland China due to its “One Child Policy” and the fact that the subjects had children already (Ding et al., 2012). This question was also dropped from our EFA results, of which 95% had child adequacy. A study from Brazil had questionable internal consistency in the EWB domain, which is explained by the level of understanding of question ge3: “I am losing hope in the fight against my illness,” which was not understood by the patients (Fregnani et al., 2013). However, this question was still in our EFA result.

Regarding the structure of the questionnaire: the convergent validity results of the Thai Modified FACT-CX tended to correlate moderately with WHOQOL-BREF. The previous study compared FACT-CX with medical outcomes, 39-items, and the Short-Form Health Survey (SF-36). Only FWB and EWB had significant correlations with the domains in SF-36 (Fregnani et al., 2013). Comparing the structure of FACT-G with a study conducted in Hong Kong cancer patients found that the subscale correlations between the FACT-G and the WHOQOL-BREF tended to be low. The authors explained the result by noting that the 2 quality of life questionnaires were interested in different aspects. Only FACT-G focuses on cancer treatment (Yu et al., 2000). The study of FACT-G in cervical cancer patients used multiple questionnaires to supplement the correlation results (Ashing-Giwa et al., 2008). 

The discriminant validity in our study shows the same differentiation pattern of quality of life score between WHOQOL-BREF and the modified FACT-CX, except aged more than 60 yr. This finding may be as a result of the weak point of the Thai WHOQOL-BREF, which limits using age less than 60 years (Mahatnirunkul et al., 1998). In other results, the previous FACT-CX study shows an ability to differentiate perceived health status and ECOG performance. But the FACT-CX score could not differentiate stage (Ding et al., 2012; Fregnani et al., 2013). The same discriminative results between the Modified FACT-CX and WHOQOL-BREF in our study may be from untreated patients with side effects that could not be seen.

The majority of the subjects were untreated patients with locally advanced cervical cancer, a primary school education, economic problems, who were unemployed. Referring to the situation reported in 2017, more than 50% of patients did not receive radiotherapy as indicated in Mainland China, and only 6 of 11 countries in Southeast Asia had facilities to treat cervical cancer (Wang et al., 2017; Calaguas and Gubat 2017). Thus, a number of patients had no access to treatment. Our study may imply that cervical cancer is a disease of low socioeconomic people, which is similar to findings from Brazil. Thus, we should be careful measuring the quality of life in cervical cancer patients when using non-factor analysis validated instruments. 

There are some limitations. First, this study included only untreated, locally advanced cervical cancer patients. Therefore, there were no patients with radiotherapy side effects included. Second, our participants were of different religions. Belief about disease may influence quality of life. A future study comparing the results with treated patients should be conducted. Finally, the Thai modified FACT-CX was found to be both reliable and valid for measuring quality of life in untreated cervical cancer patients.
